# Editorial: Silver fluoride and caries management

**DOI:** 10.3389/froh.2024.1480767

**Published:** 2024-09-03

**Authors:** Catherine Abdelsayed, Riwa Deghaim, Danaé Dimitracopoulos, Lucia Momblona Robres, Raman Bedi

**Affiliations:** ^1^ Edinburgh Medical School: Molecular, Genetic and Population Health Sciences, Usher Institute, The University of Edinburgh, Scotland, United Kingdom; ^2^ Higher Institute of Public Health, Saint Joseph University of Beirut, Beirut, Lebanon; ^3^ Faculty of Biology and Medicine, University of Lausanne, Lausanne, Switzerland; ^4^ General Health Psychology, International University of Valencia, Valencia, Spain; ^5^ King's College, London, United Kingdom

**Keywords:** dental caries, silver diamine fluoride, silver fluoride, caries activity, prevention

**Editorial on the Research Topic**
Silver fluoride and caries management

If the public was asked to list diseases that have the highest global burden, oral diseases may not be included in the provided answers. According to the WHO (1) in the report on global oral health status, oral diseases represent a global public health problem that necessitates intervention. Since 1990, oral diseases have been the most prevalent worldwide condition. In 2019, 3.5 billion people were affected globally by oral diseases. Moreover, there is an alarming proportion of affected people in middle-income countries where 3 out of 4 residents are affected. Accordingly, oral diseases not only affect the individual's health and well-being but also affect healthcare systems and economies. This can be shown by the US$ 387 billion spent by 194 countries in 2019 as a direct cost for oral diseases in addition to global US$ 323 billion productivity losses resulting from the same conditions.

One of the oral diseases of the highest-burden is untreated dental caries, whether in deciduous or permanent teeth. More than 30% of humanity lives with untreated dental caries. 2 billion people globally have untreated caries in their permanent teeth compared to 514 million children with untreated caries in deciduous teeth making it the sole most frequent chronic childhood disease. The estimated global average prevalence of untreated caries in deciduous and permanent teeth are 43% and 29% respectively. Although these numbers are alarming, it is possible to reduce the global disease burden by self-care or simple measures (1).

The WHO recognized the important link between oral health and overall health, where dental caries and other oral diseases can have significant consequences impacting nutrition, speech, self-esteem, and overall quality of life (2). Accordingly, WHO included a dental section in its Essential Medicines list.

The WHO Essential Medicines List is a curated compilation of the most effective, safe, and cost-effective medicines needed for basic healthcare (3). It provides a guiding framework for countries to prioritize access to essential medicines while ensuring their availability and affordability (4). This list is extremely important because it influences national pharmaceutical policies and procurement decisions, eventually affecting the delivery of healthcare services.

The inclusion of a dental section in the WHO Essential Medicines List highlights the important link between oral health and overall health. In a significant step forward, the World Health Organization has added silver diamine fluoride (SDF) to the dental section of its Essential Medicines List in 2021. This shows the WHO's recognition of its potential to revolutionize the prevention and management of dental caries, especially in resource-constrained settings and vulnerable populations like the elderly and disabled.

According to WHO (5), the newly added medicine, SDF, Ag F(NH3)2 is a clear liquid formulation containing high concentrations (most commonly 38%) of fluoride (approximately 44,800 ppm) and silver (approximately 253,870 ppm silver) (6). It has garnered attention for its remarkable efficacy in preventing and arresting dental caries, showing an 81% success rate for a topical application on primary teeth (7). 80% effectiveness is also confirmed as the general efficacy rate (5). The material costs of recommended bi-annual applications are approximately USD$0.20 per year per person.

The decision of the EML revising committee to add SDF was not only based upon its numerous advantages but also due to the strong 50-year existing evidence about its effectiveness. Studies proved that SDF application decreased the incidence of new dentinal caries significantly when compared to placebo, no received treatment, and fluoride vanish in a 2-year follow-up. SDF was also found to successfully arrest root caries by 90% at 30-month follow-up. Another advantage is that SDF has no reported side effects. However, aesthetically, it stains the treated caries with a dark color. Tooth pain and irritation of the gingiva have rarely occurred and if occurred, they subsided quickly (5).

The importance of SDF extends beyond its role in addressing the global burden of dental caries. For aging populations and individuals with disabilities, SDF offers a minimally invasive, cost and time-effective alternative to traditional restorative treatments. Decades of research have demonstrated its ability to halt the progression of carious lesions, particularly in aging populations or populations with limited access to conventional dental care.

Aging populations often face unique challenges when it comes to maintaining oral health. As people age, they may experience decreased manual dexterity, making it difficult to maintain proper oral hygiene. In addition, many medications commonly prescribed to older adults can cause dry mouth, which could increase the risk of dental caries (8).

Similarly, people with disabilities may face barriers to accessing dental care due to physical limitations (9), transportation difficulties (9), or financial constraints (10). SDF can be easily applied in non-traditional settings, such as nursing homes or community health centers, thus making it more accessible to this population (11). By preventing and arresting dental caries, SDF can help reduce the need for more invasive and expensive dental treatments, which may be especially challenging for people with disabilities.

In this comprehensive discussion, we explore the diverse ramifications of integrating SDF into worldwide oral health strategies, emphasizing its potential to mitigate socioeconomic disparities associated with dental caries. We stress the necessity of proactive measures in fostering oral health equity, advocating for collaborative endeavors and sustained investment in accessible, evidence-based interventions.

Ultimately, this editorial underscores the imperative of addressing the global caries crisis through multifaceted approaches, highlighting SDF as a promising solution to not only prevent and treat tooth decay but also to narrow dental care gaps and advance equity in oral health. Through concerted efforts, we envision a future where dental caries cease to pose a significant threat to global well-being.
